# Research Advancements in Peanut Proteins, Their Allergenic Potentials, and the Approaches to Mitigate Peanut Allergenicity

**DOI:** 10.3390/nu17193078

**Published:** 2025-09-27

**Authors:** Jianmei Yu, Mahshid Eghbali

**Affiliations:** Food and Nutritional Sciences, Department of Family and Consumer Sciences, North Carolina Agricultural and Technical State University, Greensboro, NC 27411, USA; meghbali@aggies.ncat.edu

**Keywords:** peanut allergy, allergenic proteins, IgE-epitope, mitigation, challenges

## Abstract

With increasing interest and demand for plant protein-based foods, the allergenicity of plant proteins has been placed in a very important position. Among plant food allergens, peanuts have been considered the most potent because peanuts often cause severe allergic reactions, even life-threatening anaphylaxis. It is well-known that allergenic proteins in peanuts trigger peanut-induced allergic reactions through binding to the immunoglobulin E (IgE) antibodies in the patients sensitive to peanuts. So far, eighteen peanut allergens have been identified. These allergens belong to different protein superfamilies with distinctive characteristics and allergenic potentials. Due to the rapid rise in peanut allergy prevalence in the past few decades, many studies have been conducted to reveal the effects of primary structures (epitopes) and conformational structures of peanut proteins on their allergenicity and to explore methods and strategies to mitigate peanut allergenicity and peanut allergy. This comprehensive review highlights the nutritional value of peanut protein related to its amino acid composition, the current prevalence of peanut allergies and its impacts on quality of life, the recent research findings on the characteristics and allergenic potential of individual peanut allergens, and the potential and challenges of different approaches and methods for mitigating peanut allergenicity.

## 1. Introduction

Consuming plant proteins offers numerous health benefits, contributing to overall well-being. Plant-based protein sources are often rich in fiber, vitamins, and minerals, while typically being lower in saturated fat and cholesterol compared to animal proteins, which can reduce the risk of cardiovascular disease [1]. Additionally, diets high in plant proteins have been associated with a lower risk of type 2 diabetes and certain cancers [2]. Furthermore, incorporating a variety of plant proteins in diets can ensure a diverse intake of essential amino acids, supporting muscle health and overall bodily functions. The high fiber content in many plant-based protein sources also promotes gut health, aiding in digestion and maintaining a healthy microbiome [3]. However, allergenicity is one of the major concerns of many plant proteins. The allergenic potential of proteins varies significantly across different plant origins, with certain families of proteins being more prone to triggering allergic reactions in susceptible individuals [4]. Peanuts are among the most potent food allergens, causing severe reactions and even anaphylaxis [5]. The allergy caused by other plant proteins may be outgrown as individuals grow up, but the peanut and tree nut allergies often last for a lifetime, because only 20% of individuals outgrow peanut allergies. Thus, a peanut allergy is the major limiting factor for the application of peanuts and peanut protein as food ingredients due to the fear of accidental exposure to allergic patients, which is the main cause of peanut allergy-related hospitalizations [6]. The pre-harvest approaches include conventional breeding and genetic modifications such as RNA interference (RNAi) and CRISPR gene editing [7,8,9]. Post-harvest approaches include physical treatments, chemical modification, and biotreatments such as enzymatic treatment and fermentation. Each method has its advantages and limitations. More importantly, these approaches reduced the allergenicity of peanuts or peanut protein to varying degrees but cannot eliminate it. Thus, more research is needed to develop innovative technology to minimize the allergenicity of peanut proteins. The purpose of this review is to update the global peanut allergy prevalence, its economic burden, its impact on the quality of life, the research advancements on allergenic peanut proteins, to highlight the methods and strategies for peanut allergenicity mitigation, and the future research needed.

## 2. Nutritional Value of Peanuts and Peanut Protein

### 2.1. Nutrient Composition of Peanuts and Peanut-Derived Products (Nutritional Value of Peanuts and Peanut Protein)

The peanut (*Arachis hypogaea* L.), also called a ground nut, is classified as a legume and an oilseed. It is an energy-dense and nutrient-balanced grain. Shelled raw peanut contains 42.64–57.47% of lipids, 3.8–7.99% of moisture, 19.19–26.58% of protein, 2–3% of ash, and 11.54–19.65% of carbohydrates with high dietary fiber (8.5%) and low sugar (4.7%) content [10]. The concentrations of these nutrients vary with the variety/cultivar and kernel size of peanuts, as well as growing location and climate. In addition, peanuts contain a significant amount of phytochemicals such as polyphenols, resveratrol, and phytoestrogens [11,12]. Peanuts are a good source of protein, healthy lipids (mainly monounsaturated), dietary fiber, vitamin E, vitamin B, minerals, polyphenols, and phytosterols [13]. Peanuts contain significant amounts of leucine, isoleucine, valine, lysine, phenylalanine, threonine, and histidine but are slightly deficient in the sulfur-containing amino acids methionine and cystine as well as lysine [14,15]. However, the essential amino acid content of peanuts and peanut-derived products is affected by peanut processing (Table 1). Roasting reduced the contents of all amino acids as a result of Maillard browning [16]. In the peanut flour, the essential amino acid contents increased with the degree of defatting. The fat-free peanut flour contains 50% or more protein, and its essential amino acids composition is similar to roasted soybean, which also contains about 50% protein [10] as shown in Table 1. In addition, peanut proteins significantly contribute to the desirable color and flavor of roasted peanuts by acting as a key precursor in the Maillard browning reaction, which occurs between protein/free amino acids and sugar during roasting. The Maillard reaction generates volatile compounds like pyrazines, furans, and aldehydes that are essential to the characteristic nutty, roasted aroma and taste through interactions with sugars [17] at the cost of essential amino acids, free amino acids, and sugar [16]. Further, it also contributes to the light or dark brown color of roasted peanuts due to the formation of brown polymers called melanoidins [18]. Overall, roasting increased the palatability and digestibility of peanuts, but prolonged roasting reduces the nutritional value of peanut protein due to the loss of essential nutrients such as amino acids and sugar.

### 2.2. Health Benefits of Peanut Consumption

Both prospective and case–controlled studies have found that consuming peanuts (not peanut butter) and tree nuts are positively associated with the reduced risks of cancers [19,20,21], cardiovascular diseases (CVD) [22], overweight/obesity [23], type-2 diabetes [24], and the mortalities caused by various diseases [25,26,27]. Regularly consuming peanuts or peanut butter also contributes to improved cognitive health in the elderly and young adults [28,29]. These health benefits are due to the fact that peanuts are packed with essential nutrients, including arginine-rich protein, heart-healthy monounsaturated fatty acids, dietary fiber-rich carbohydrates, important minerals, B vitamins, choline, resveratrol, and phytosterols [13,30]. Peanuts are widely consumed as peanut butter and roasted peanut snacks for their pleasant flavor and nutritional value in Western countries. In the United States, the total peanut consumption in 2024 is 2,198,000 metric tons, with 59% consumed as peanut butter, followed by peanut candy (17%) and peanut snacks (16%) and only 2% as an ingredient [31]. One of the major factors limiting the application of peanuts and their protein in food product development is the risk of accidental exposure of peanut allergic individuals to the peanuts, which is the main cause of peanut allergy-related hospitalizations [6]. Therefore, the application of peanuts or peanut flour in food product development also raises safety issues that have to be addressed through different strategies, including technical approaches to reduce or eliminate the allergenicity and peanut allergy management/treatment.

## 3. Peanut Allergy

### 3.1. Prevalence of Peanut Allergy

Peanut allergy prevalence exhibits significant global variation, influenced by geographical, dietary, and cultural factors (Table 2). A retrospective cohort study conducted at 37 children’s hospitals in the U.S. between 2007 and 2012 found that thes peanut was responsible for 37% of food allergy anaphylaxis cases and 35% of hospital admissions [32]. Recent studies have revealed that 2.2% of children under 18 and 1.8% of adults are allergic to peanuts in the U.S. [33,34], almost doubled from that reported a decade ago [35]. A study, which surveyed 5615 children in Israel and 5171 in the UK, found that the prevalence of peanut allergies among Jewish school children in Israel was 0.17% (8 out of 4657) compared to 1.85% (73 out of 3943) in the UK. That is, the risk of peanut allergy in the UK was more than 10 times higher than in Israel [36]. Researchers attributed this difference to dietary habits, particularly the early and frequent consumption of peanuts in Israel, where infants aged 8 to 14 months consumed a median of 7.1 g of peanut protein per month, while UK infants consumed none [36]. Currently, around two-thirds of schools in Europe have at least one child at risk of anaphylaxis due to peanut allergies [37]. Additionally, European countries display marked heterogeneity in peanut allergy prevalence. For instance, systematic reviews indicate that lifetime prevalence rates across Europe range significantly, from as low as 0.1% to as high as 6% [38]. Notably, Northern European countries generally report lower incidences in contrast to those in Southern Europe, suggesting that dietary customs and nutrient availability influence these outcomes. In Ireland, one study involving 1421 Irish children found that 1.69% of 2-year-olds had a confirmed peanut allergy [39]. In countries like France and Belgium, patterns of allergy development show nuanced differences, with early peanut introduction often correlating with better prevention outcomes compared to countries where such practices are less common [40]. In the Netherlands, the number of people with a peanut allergy increased in the period from 1995 to 2007. Conversely, in Australia, the prevalence of peanut allergies in infants has reached around 3%, with notably heightened rates observed among infants of Asian descent [41].

In Asia, peanut allergies are relatively uncommon compared to Western nations, although regional variations exist. For example, in Japan, wheat allergies are more predominant, while shellfish allergies are more common in Singapore and the Philippines [59]. The prevalence of peanut allergies in Thailand is reported to be very low, 0.1–0.3% among children 1–4 years old [57]. In China, although the prevalence of peanut allergies is low (0.2–0.3%) compared to Western countries, there is a growing trend of emerging peanut allergies, particularly in major cities [48,49]. Urbanization is a notable contributing factor to the increase in allergic conditions due to changes in lifestyle and environmental exposures [60,61]. Similarly, in South Africa, studies have reported food allergy prevalence rates ranging from 2.5% in an unselected population of children to 40% in children with moderate to severe atopic dermatitis [62]. These trends suggest that peanut allergies may become more prevalent globally, affecting both developed and developing nations.

### 3.2. Economic Burden of Peanut Allergy

Peanut allergies are one of the most severe food allergies. Peanuts can cause a severe, potentially life-threatening allergic reaction (anaphylaxis). Because of the high prevalence and high percentage of anaphylaxis, peanut allergies can result in significant medical, out-of-pocket, and opportunity costs to payers, parents, and employers [63]. The economic burden of peanut allergies include direct and indirect costs. The direct costs include medical care such emergency room visits, hospitalizations and ongoing treatments, medication such as epinephrine auto-injectors (EpiPens) and other medications to manage allergic reactions, and food challenge for diagnosis. The indirect costs include lost productivity due to missed work or school days, the need for specialized allergen-free foods, which are often more expensive, and the stress of dietary vigilance, further diminishing the quality of life and increasing psychological distress for patients and their families [64]. Patients with a peanut allergy-related diagnostic code incurred almost double all-cause health care costs vs. controls (USD 6436 vs. USD 3493, *p* < 0.001), mainly from inpatient and outpatient medical costs (USD 5002 vs. USD 2832, *p* < 0.001) [65]. Pediatric patients in the United States with peanut allergy and reactions triggering HRU had significantly higher comorbidity burdens, HRU, and direct health care costs regardless of asthma-related costs versus those without a peanut allergy [64]. Hence, it is important to improve the allergenic safety of peanuts to protect those who are sensitive to peanuts.

### 3.3. Impact of Peanut Allergy on Quality of Life

Peanuts can lead to severe, potentially fatal, allergic reactions, which further exacerbate the negative impact on the quality of life of affected individuals and their families [66,67]. Peanut allergies typically manifest initially during childhood between 4 months and 2 years of age [68]. However, some adults develop a peanut allergy. For instance, a study based on surveys and clinical records in the United States and Australia found that about 10–15% of adults developed a sensitivity to peanuts in their adulthood [34]. The burden of a peanut allergy is not only physical but also psychological, affecting daily life and causing stress and anxiety [64,69]. The restrictions imposed by peanut allergies, such as dietary limitations and hypervigilance, lead to an impaired quality of life [70,71]. The fear of accidental ingestion and the need for constant vigilance contribute to the negative impact on quality of life [72]. Moreover, peanut allergies are typically lifelong for many individuals, although some children do outgrow the allergy. About 20–22% of children with peanut allergies may eventually outgrow the allergy by their teenage years [73], with the chances of outgrowing the allergy being higher for those with milder reactions and lower levels of peanut-specific IgE antibodies.

## 4. Characteristics of Allergenic Proteins in Peanuts

Many studies have been conducted to characterize the specific peanut proteins responsible for peanut allergies. It was reported in 2012 that all known allergenic peanut proteins together comprise approximately 85% of the total protein content, with Ara h 1, Ara h 2, and Ara h 3/4 accounting for a combined 75% [74]. At that time, only 11 peanut allergens were identified, and this number increased to 13 in 2013, 17 in 2015, and 18 in 2021. In addition, most of the peanut allergens have two or more isomers. Table 3 includes the officially registered peanut allergens and their isomers [75]. Therefore, the allergens in peanuts may be more than 85% of the total proteins. These allergens are categorized by their structural families, which include cupins, 2S albumins, Profilins, PR-10 proteins, non-specific lipid transfer proteins (nsLTP), oleosins, defensins, and cyclophilin, respectively. As shown in Table 3, these allergens differ in molecular weight and the length of the amino acid sequence. From the GenBank Protein number, we obtained the protein sequence of each allergen and counted the number of individual amino acids in the allergen using the AI tool DeepSeek, and the results are presented in Table 4. This section describes the characteristics of identified peanut allergens based on the protein families they belong to due to their similarities in molecular structures, amino acid composition, and solubility.

### 4.1. Peanut Cupins

The Ara h1 and Ara h3 belong to the cupin superfamily. They are the dominating peanut storage proteins. The Ara h 1 is vicillin-type 7S globulin and forms a stable symmetrical trimmer with a 3-fold axis running between the monomers, while Ara h 3 is a legumin-type 11S globulin, also known as glycinin, and can form hexameric complexes [76]. Ara h 4 is actually an isomer of Ara h 3, and it was renamed as Ara h 3.02 by the WHO and International Union of Immunological Societies (WHO/IUIS) Allergen Nomenclature Sub-committee in 2012. Ara h 3 consists of several polypeptides with MW about 14–45 kDa that can be classified as acidic and basic subunits [77]. Ara h 1 and Ara h 3, together with Ara h 2, account for approximately 75% of the total protein in peanuts [74]. Ara h1 and Ara h3 share a 21% sequence identity. Ara h 1 and Ara h 3 are rich in polar and charged amino acids with glutamic acid, glutamine, aspartic acid, asparagine, leucine, and arginine being most abundant (Table 4), which makes them soluble in water. The optimal pHs for solubilizing Ara h 1 and Ara h 3 are similar (pH 7–10), but their isoelectri points (IP) are different [78,79,80,81]. The IP of Ara h 1 and Ara h 2 were reported to be 4.55 [81] and 5.2 [78], respectively. This indicates that Ara h 3 cannot be completely precipitated at the IP of Ara h 1 and vice versa. In addition, studies have illustrated that both Ara h 1 and Ara h 3 tend to form insoluble aggregates or oligomers following thermal processes such as boiling, roasting, and frying, and therefore may not be detected in food products with methods depending on allergen solubility, such as ELISA [82]. However, they can be detected by gel electrophoresis (reduced SDS-PAGE and Western blot).

### 4.2. Peanut Conlutins (2S Albumins)

Peanut conlutins include Ara h 2, 6, and 7. They belong to the prolamin superfamily, a type of seed storage protein, and they are all 2S albumins with molecular weights lower than globulins (Ara h 1 and Ara h 3). The 2S albumins are the second most abundant proteins in legumes, accounting for 10–30% of the total proteins [83]. Ara h 2 and Ara h 6 share a 59% amino acid sequence identity, and Ara h 2 and Ara h 7 share a 42% amino acid sequence identity. However, a more recent study reported that Ara h 6 and Ara h 7 shared a 77% and 60% homology with Ara h 2, respectively, but not all epitopes identified in these conglutins were shared among the three allergens [84]. They are characterized by a high content of cysteine residues and compact, stable structures, other abundant amino acids in these peanut allergens being glutamic acid, aspartic acid, leucine, and arginine, as shown in Table 4. Thus, they are soluble in an aqueous buffer within a broad pH range (2–10) and can be precipitated at pH 5.2 (Ara h 2 and 6) and 5.7 (Ara h 7) [78,85,86,87]. Ara h 2 exists in two isoforms, Ara h 2.02 and Ara h 2.01, with MW 16.67 and 18.05 kDa, respectively, while Ara h 6 and Ara h 7 are smaller and have a MW of 15 kDa (Table 3).

### 4.3. Peanut Profilin (Ara h 5) and PR-10 Protein (Ara h 8)

Ara h 5 is a member of a Profilin allergen family and structurally similar to pollen allergens, such as birch allergen Bet v 2 and latex allergen Hev b 8 [88]. Ara h 8 (MW = 18.2 kDa) is found in small quantities in peanuts and is a protein belonging to the PR-10 family, which is a pathogenesis-related protein with a protective role in the innate immune system of plants [89]. They are soluble at neutral to slightly alkaline pHs (pH 6–8). The isoelectric point (IP) of Ara h 5 is pH 4.6 [88], while the IP of Ara h 8 has not been explicitly stated yet, but it is estimated as pH 5.03 [90]. The amino acid compositions derived from their sequences show that Ara h 5 is a small protein (MW = 10–15 kDa) rich in leucine, valine, lysine, and glutamic acid but lacks cysteine, while Ara h 8 is rich in glycine, leucine, glutamic acid, lysine, and valine but also lacks cysteine and tryptophan (Table 4). The lack of cysteine means that they do not have disulfide bonds, which may contribute to the instability of Ara h 8 during peanut roasting and gastric digestion [91].

### 4.4. Non-specific Lipid-Transfer Proteins in Peanuts

Ara h 9, Ara h 16, and Ara h 17 are non-specific lipid-transfer proteins (nsLTPs). The nsLTPs are small proteins characterized by a tunnel-like hydrophobic cavity, which makes them suitable for binding and transporting various lipids [92]. They are found in all plants and are widely known for their antimicrobial activities (including antibacterial, antifungal, and antiviral) because they can cross fungal and bacterial membranes, generating pores that cause the efflux of cell electrolytes and eventually cell death [93,94]. The Ara h 9 and Ara h 17 are type 1 (nsLTP-1), while Ara h 16 is a type 2 non-specific lipid-transfer protein (nsLTP-2). They have different molecular weights and amino acid sequence lengths. Ara h 9 is rich in the amino acids alanine, leucine, and glycine, while Ara h 16 and 17 are rich in proline, cysteine (Ara h 17), and serine in addition to alanine, leucine, and glycine [75] (Table 4). Ara h 16 and Ara h 17 have a 35% identity to Ara h 9 and 62% identity to Ara h 9, respectively [95]. They are homologous to Pru p 3, an allergenic protein of peaches, meaning they share a high degree of structural and functional similarity and can lead to cross-reactivity [95,96].

### 4.5. Peanut Oleosins

Oleosins are structural proteins found in plant oil bodies (oleosomes) that are crucial for oil storage, stability, and mobilization during seed germination [96,97]. Peanut oleosins include Ara h 10, 11, 14, and 15. These allergens contain a high proportion of hydrophobic amino acid residues such as proline, glycine, alanine, leucine, isoleucine, and valine (Table 4), thus having a long central hydrophobic stretch, which makes them have low solubility in an aqueous solution, but they are soluble in organic solvents such as chloroform/methanol [98,99]. The IPs of Ara h 10 and Ara h 11 were reported to be pH 9.36–9.6 and 10.08, respectively [87], while the IPs of Ara h 14 and Ara h 15 were pH 4.5–6.0 [100]. This suggests that the regular protein extraction procedure using phosphate buffer cannot extract these allergens unless a sufficient surfactant (such as tween 20 or tween 80) is added, and Ara h 11 and Ara h 12 cannot be precipitated in the protein isolate-producing process where pH 4.5 was used to precipitate proteins. Peanut oleosins isolated from in-shell roasted peanuts have been reported to cause allergic reactions in a clinical study with severe allergic symptoms [99]. The sequence identity between Ara h 15 and Ara h 10 or Ara h 11 is below 48%, and the sequence identity to Ara h 14 is below 30% [75].

### 4.6. Peanut Defensin Proteins

The defensin proteins are small cysteine-rich proteins involved in the plant’s defense mechanisms. Ara h 12 and Ara h 13 are two defensin proteins isolated from lipophilic peanut extract. They contain abundant cysteine, glycine, and arginine, which are common in defensin proteins, as well as hydrophobic amino acids leucine and valine (Table 4). The Ara h 13 has two isomers with similar molecular weights around 11 kDa (non-reduced) and a 94% identity, while Ara h 12 is 27 amino acids different from Ara h 13, and they share a 43% identity [100]. Due to the high cysteine content, the amino acid residues in defensin proteins are held by four disulfide bonds [101], which contribute to the stability of defensin proteins. Plant defensins act as natural antimicrobial peptides against different pathogens like viruses, bacteria, and fungi [102]. Peanut defensins at a concentration of 25 to 100 mg/mL showed a prolonged inhibitory effect on the growth of fungi Alternaria and *Cladosporium* species and slightly reduced the growth of *F. culmorum* and *A. flavu*, two mycotoxin-producing fungal species [103].

### 4.7. Peanut Cyclophilin-Ara h 18

Ara h 18 is the most recently identified allergenic protein in peanuts [75]. It has a molecular mass of 18.2 kDa and is a cyclophilin protein with peptidyl-prolyl cis-trans isomerase activity. The abundant amino acids in Ara h 18 are glycine, followed by valine, serine, threonine, phenylalanine, alanine, and lysine (Table 4). Cyclophilins are known for their high-affinity binding to the immunosuppressive agent cyclosporine A [104]. They are highly conserved and have been reported as IgE binding proteins in grass, tree, and weed pollen, several plant foods such as peanuts, carrots, pumpkins, and tomatoes, as well as in several fungi and house dust mites [104,105]. Ara h 18 was identified as a minor component of the peanut protein extract. Its amino acid sequence, as determined by LC-MS/MS, matched peanut cyclophilin, and its molecular length, estimated based on the sequence, was 171 amino acid residues with MW of 18.2 kDa and an isoelectric point of pH 8.4 [104].

Based on above information, it is clear that (1) peanut allergenic proteins that belong to different protein families with specific biological functions during the peanut development and maturing process; (2) the allergenic proteins in the peanuts differ in molecular weights, solubilities and isoeletric points, thus the extraction, purification and isolation of these proteins need to be conducted at different pH and using different techniques; (3) as research continually progress, new allergenic peanut proteins may be discovered in the future.

## 5. Allergenicity of Peanut Proteins

The review of Ozias-Akins & Beiteneder provides excellent insights into the relationship between the allergenicity of peanut proteins and their biological functions [106]. The peanut plant flowers aboveground but produces pods with peanut kernels inside underground. This exposes pods and kernels to soil-borne pathogens and pests during maturation. When pathogens or insects invade the pods and kernels, the peanut proteins interact with pathogens or pests, modify/damage their cell membranes, modulate signaling pathways, and bind to immune receptors, leading to specific IgE production [106]. Therefore, it is reasonable to say that peanut allergenic proteins are synthesized as part of the defense system of peanut plants against soil pests and microorganisms such as bacteria and fungi. Due to the similarity in stability and allergenicity of peanut proteins in the same protein family, the allergenicity of allergens in the same protein family is described together in this review.

### 5.1. Allergenicity of Peanut Cupins

Peanut cupins, specifically Ara h 1 (a vicilin) and Ara h 3 (a glycinin), are major peanut allergens. Ara h 1 is particularly notable for causing a significant number of severe, potentially fatal, anaphylactic reactions in patients. Ara h 3 is resistant to enzymatic breakdown [107,108]. Both Ara h 1 and Ara h 3 are known to be relatively heat stable, indicating that thermal processing such as roasting, frying, or boiling cannot completely eliminate their allergenic potential. The basic subunits of Ara h 3, and to a lesser extent the acidic subunits, bind IgE and might act as allergenic peptides [77]. Furthermore, Ara h 1 and Ara h 3 usually form high-molecular-weight protein oligomers (specifically trimers and hexamers) during thermal processes such as roasting [74]. The oligomeric structures of the purified Ara h 1 and Ara h 3 demonstrated strong IgE-binding properties [109,110].

### 5.2. Allergenicity of Peanut 2S Albumins

Both in vitro and in vivo studies have provided strong evidence that the 2S albumins are the most important allergens in peanuts, with Ara h 2 and Ara h 6 being the most potent and well-studied peanut allergens [77,111,112,113,114]. An earlier study reported that Ara h 2 was the most frequently recognized allergen by peanut-sensitive patients, followed by proteins smaller than 15 kDa [77]. Studies have demonstrated that native Ara h 2 and Ara h 6 have the same or similar allergenic potency [111,114]. A cohort study with 100 children found that most patients with a peanut allergy were sensitized to both Ara h 2 and Ara h 6, and Ara h 2 induced allergen-specific IgE binding more than Ara h 6 did, as shown by the ImmunoCap measurement, despite their 60% sequence similarity [112]. The presence and levels of specific IgE antibodies to Ara h 2 and Ara h 6 could provide the greatest accuracy in diagnosing peanut allergies in Component-Resolved Diagnostics [115]. The Ara h 7 has three isomers, and Ara h 7.0201 was reported as the most frequently (80%) among the three and was as allergenic as Ara h 2 and Ara h 6 in inducing basophil degranulation [116], although Ara h 7 accounts for only 0.5% of the total peanut protein [117]. Therefore, the peanut albumins are considered the most potent allergens among peanut proteins. Ara h 2 and Ara h 6 have been reported to be highly stable to heat treatment and resistant to protease digestion because of their tightly coiled structure stabilized by multiple disulfide-bridged cysteine residues, which are considered to be contributing to their strong allergenicity [111]. The stability characteristics of Ara h 7 are still under research.

### 5.3. Allergenicity of Ara h 5 and Ara h 8

Both Ara h 5 and Ara h 8 were stated as minor peanut allergens involved in pollen-associated peanut allergies. They predominantly contribute to the development of pollen-associated allergic reactions due to cross-reactivity with their homologues in birch and grass pollen [118]. A recent study revealed that the allergic reactions caused by Ara h 5 and Ara h 8 are different. The results from in vitro immunoreactivity tests with serum samples from 10 peanut allergic patients and 27 other allergic patients, mast cell degranulation assay, and an in vivo study with sensitized mice show that Ara h 5 resulted in a stronger IgE binding and the ability to induce ß-hexosaminidase release, causing more severe lung inflammation than Ara h 8. Meanwhile, Ara h 8 caused more severe intestinal inflammation than Ara h 5 [119]. This study illustrates that the conformational epitope of the Ara h 5 protein was crucial in the process of sensitization. Ara h 8 was found in small quantities in peanuts and was unable to withstand heat and digestive enzymes and pH in the stomach, thus it was considered a minor food allergen responsible for oral allergy syndrome, in which sensitization to airborne allergens causes a type 2 allergic reaction to ingested foods [89,120].

### 5.4. Allergenicity of Peanut Non-Specific Lipid Transfer Proteins (nsLTPs)

The nsLTPs Ara h 9, Ara h 16, and Ara h 17 in peanuts have been identified as potential allergens. A recent study identified nine linear IgE-binding epitopes in the Ara h 9 using serum IgE samples from 55 peanut-allergic individuals in the US and 17 patients from Spain, and similar IgE-binding profiles for Ara h 9, Jug r 3, and Pru p 3 were identified by the US and Spain serum samples [121]. This indicates that Ara h 9 is an allergenic protein not only important to Mediterranean people but also to the US population. The Radioallergosorbent Test (RAST) test found that Ara h 16 and Ara h 17 bound to the serum IgE of 4 of 25 clinically defined peanut-allergic subjects and one peach-allergic subject. These two nsLTPs were accepted by the WHO/IUIS Allergen Nomenclature Sub-committee [75], but the respective papers have not yet been published.

### 5.5. Allergenicity of Peanut Oleosins

The peanut oleosins Ara h 10, 11, 14, and 15 are considered marker allergens for severe reactions. Therefore, they contribute to the overall allergenicity of peanuts. A study evaluated the allergenicities of purified mixtures of Ara h 10/11 and Ara h 14/15 obtained by preparative immunoblot and basophil activation tests (BAT) using sera from 52 peanut-allergic individuals suffering from a severe systemic reaction and found that more than 65% (35 of 52) of these subjects were sensitized to peanut oleosins [99]. It was reported that in-shell peanut roasting significantly enhanced the allergenicity of oleosins [122]. These oleosins have been proven to be clinically relevant peanut allergens, and they are likely associated with severe allergic symptoms [104], and they contribute to the overall allergenicity of peanuts, although it is unknown if they are as allergenic as peanut albumins. More comparative studies are necessary to elucidate their allergenic potential.

### 5.6. Allergenicity of Peanut Defensins

Identified peanut defensins include Ara h 12 and Ara h 13. The study on the allergenicity of Ara h 12 and Ara h 13 is limited. These two lipophilic proteins only bind to the IgE antibodies of patients severely allergic to peanuts [100], indicating their weak allergenicity. Their specific epitopes responsible for the immunoreactivity have not been reported yet. More studies with peanut-allergic populations in different parts of the world may provide more insights into the allergenicity of peanut defensins, as the immunological responses to the same peanut allergen vary greatly with populations and countries.

### 5.7. Allergenicity of Peanut Ara h 18

Ara h 18 was reported to be responsible for the peanut allergy in the population with positive ImmunoCAP test results to whole peanuts (3.1–48.4 kUA/L) but negative ImmunoCAP test results (<0.35 kUA/L) to major peanut allergens Ara h 1, 2, 3, 6, 8, and 9 [104]. The cyclophilin Ara h 18 has displayed an allergenicity similar to peanut lipid transfer proteins (LTPs) and defensins but weaker than oleosins and storage proteins [104,123]; thus, it has been considered a minor allergen. By the date this review is completed, the specific IgE epitopes of Ara h 18 have not been reported.

### 5.8. Cross-Reactivity of Peanut Allergens with Other Proteins

There are cross-reactivities among peanut allergens and other plant proteins, which means that if someone is sensitive to one protein, this person may also be sensitive to one or more of the other proteins. Desensitization to different allergens is correlated with the reduced severity of the patients’ symptoms [124,125]. For example, Ara h 6 is generally considered to cross-react extensively with Ara h 2 because of their structural homologies [126]. Ara h 8 has been reported to have cross-reactivity with Bet v 1 (birch pollen), Gly m 4 from soybeans, Pru av 1 from cherries, and Fagales tree pollens and legumes of the Fabaceae family due to the high structural homology among these allergens [89]. Peanut oleosins share structural similarities with oleosins found in other seeds, such as sesame and hazelnut, which can lead to cross-reactivity. Ara h 18 has a high sequence identity (88–91%) with pollen cyclophilins in birch (Bet v 7), olive (Ole e 15), and periwinkle (Cat r 1) pollen, which has been shown to correlate with immunological cross-reactivity and might have clinical implications [104,127]. The cross-reactivity of peanut defensins with other allergenic proteins has not been reported, but it is worth investigating because there is increasing evidence that seed defensins are the causative molecules in several mugwort pollen-associated food allergies [128]. Based on the above description, the relative allergenicity of peanut allergens in different protein groups/families are summarized in Table 5.

## 6. Factors Influencing Peanut Allergenicity

The allergenicity of peanuts is influenced by the protein structure, digestibility and stability of protein, the processing methods, individual susceptibility, age and geographic location, and food matrix.

### 6.1. Peanut Protein Structure and Allergenicity

#### 6.1.1. Primary Structures and Allergenicities of Peanut Proteins

The primary structures of peanut allergens are their amino acid sequences. Peanut proteins were found to contain multiple binding sites for immunoglobulin E (IgE). These binding sites are named as epitopes, which contain different types, numbers, and sequences of amino acids. The numbers of epitopes identified within the molecules of Ara h 1, Ara h 2, Ara h 3, and Ara h 6 are 20, 26, 5, and 7, respectively [131,132]. The Ara h 2 has eight cysteine residues that could form up to four disulfide bonds, which play a critical role in stability and determine the IgE-binding epitopes [133]. Although the Ara h 3 content is high in the peanut, its allergenicity has been reported to be the lowest among these four allergenic proteins [134]. One study found that the IgE from patients with a more severe peanut allergy recognized fewer linear epitopes of Ara h 2 and Ara h 6 than did subjects with milder reactions and bound these epitopes in characteristic patterns [135]. Five IgE-binding epitopes were identified in the native and digested Ara h 1 (pepsin digestion at pH 2.5 for 2 h, followed by trypsin and chymotrypsin for 15 min at pH 6.5) [136]. The two identified epitope sequences of Ara h 5 are WETIYSR and FHWWYLK, which are particularly important for IgE binding and triggering allergic reactions in individuals sensitive to Ara h 5 [119]. The study of Ehlers and collegues identified 14 linear epitopes using peptide microarray analysis and 39 peanut-allergic patients sensitized to Ara h 7, 10 of them were able to bind both IgE and IgG4, 1 only bound to IgE, and 3 only bound to IgG4, while 3 out of 14 were unique for each isoform (Ara h 7.0101: aa 97–109; Ara h 7.0201: aa 122–133; and Ara h 7.0301: aa 65–74) but scarcely recognized by IgE [137]. Further, it was discovered that five IgE-binding sequential peptides representing the overall amino acid sequence were located in the C-terminal domain of Ara h 15, with KDRAKDY being the representative sequence, whereas the peptide DKARDVKDRAKDYAG displayed the highest IgE-binding affinity. The specific epitopes of other oleosin peanut allergens are not well-defined [99]. The IgE-binding epitopes of other peanut allergens have not been reported, and more research is needed to characterize the immunogenic epitopes in those proteins.

#### 6.1.2. Conformational Structure and Allergenicity of Peanut Protein

Peanut allergenicity is closely linked to the secondary and tertiary structures of peanut proteins. The secondary structures, especially the α-helices and β-sheets, influence the protein’s resistance to digestion and denaturation, while the three-dimensional structure of allergens can shield or protect IgE-binding epitopes from enzymatic degradation, allowing these epitopes to remain intact and trigger allergic reactions [133,138]. An early study found that the best estimates of secondary structure proportions of the native Ara h 2 were 18.2% of the molecule in α-helices, 54% in β-pleated sheets, and 27.7% in a random coil configurations. While the reduced Ara h 2 exhibits a secondary structure predominated by the β-pleated sheet (82.3%), the rest of the molecule is a random coil conformation [133]. The oligomeric forms of Ara h 1 and other peanut allergens are covalently stabilized when peanuts are cooked or roasted, which may be related to the enhanced allergenicity of peanuts [139]. This is also observed for other food allergens, as reported in a recent review, where homo-oligomerization could increase the cross-linking of B cell receptors (BCRs) on IgE-B cells and the high-affinity IgE receptor FcεRI-bound IgE on other effector cells by providing at least two epitopes [140]. Changes in these structures, induced by processing or digestion, can alter allergenicity, either reducing or enhancing it.

### 6.2. Effects of Food Matrix

The food matrix is a crucial factor influencing the allergenicity of peanut proteins. It can either enhance or reduce the likelihood and severity of allergic reactions. The food matrix influences the allergenicity of proteins in three ways: (1) affecting the protein’s structure and release from the food, (2) altering its digestion and stability, and (3) impacting how the immune system reacts to the protein [141]. One study found that increased fat content in food matrices could enhance the allergenic activity of peanut proteins, but the bio-accessibility of peanut allergens is unaffected by the dessert or cookie matrices [142]. Another study demonstrates that protein-rich food matrices such as the chocolate matrix or soy milk clearly protected allergens from pepsin digestion because the extra proteins saturated the pepsin, which could allow the intact allergens to be carried into the small intestine [143]. This may increase or decrease the likelihood and severity of peanut allergy.

### 6.3. Individual Age and Geographical Location

A multiple-institute study investigated the association between patient demographics (age, location) and allergic sensitization to peanut components Ara h 1, 2, 3, 8, and 9 across the United States by analyzing the IgE binding using serum samples from 12,155 individuals with peanut extract specific IgE levels of 0.35 kUA/L or higher [144]. The study found that among this population of peanut-sensitized individuals, 79.1% were children (<20 years old). Although sensitization was more prevalent to Ara h 2 than to the other storage proteins, a sizable fraction of patients was sensitized to Ara h 1 and/or 3 but not to Ara h 2 (eg, 13% of children < 3 years old). Moreover, 9.6% of children, 10.2% of adolescents, and 10.5% of adults were sensitized to Ara h 9, whereas 2.4% of children, 49.4% of adolescents, and 42.9% of adults produced IgE to Ara h 8 (pathogenesis-related protein 10). Sensitization to Ara h 8 alone was markedly higher in the Northeastern United States relative to other regions of the country. This study demonstrates that sensitization to individual peanut components is highly dependent on age and geographic location [144]. The higher sensitizing rate of adolescents and adults to Ara h 8 may be related to the cross-reactivity between Ara h 8 and birch pollen Bet v 1, while the sensitization to Ara h 9 may be linked to the cross-reactivity with hazel nuts and peach pollen since Ara h 8 and Ara h 9 are mild allergens [96]. Other examples on age and geographically dependent peanut allergies are shown in Table 2. Genetic predisposition, eczema, and early exposure to peanuts also play a role in determining an individual’s risk of developing a peanut allergy.

### 6.4. Effects of Peanut Processing Methods on the Allergenicity of Peanut Proteins

Thermal processing, such as roasting, boiling, and oil frying, is a key step in developing the flavor, aroma, and texture of peanuts for consumption or further processing. These processing methods can all affect the structure of peanut proteins, potentially reducing or increasing their allergenic potential. Thermal processing, particularly roasting, has been found to increase the stability of peanut proteins, potentially leading to enhanced allergenicity, while boiled and oil-fried peanuts exhibit weaker allergenicity both in vitro and in vivo (mice model) [145,146]. Studies have shown that roasting peanuts can increase the IgE-binding properties of peanut allergens, thereby potentially increasing their allergenicity [99,147]. For native peanut proteins, only a limited number of epitopes can bind to IgE because many are buried in the tertiary structure, while heat treatment results in the unfolding of the protein and exposes more epitopes to IgE antibodies [138]. Additionally, studies also found that the Maillard reaction between peanut allergens and glucose during peanut roasting resulted in the formation of advanced glycation end products (AGEs), with a higher resistant to peptic digestion and higher in vivo allergenicity of Ara h 1 and Ara h 2, as evidenced by the increased release of Th2-type cytokines, antibodies, and histamines and the increased degree of degranulation of rat basophilic leukemia (RBL) cells in the AGE-Ara h 1 group [138,148,149]. However, an in vitro study reported that after heating Ara h 2/6 (purified from raw peanuts) with glucose in a dryer for 20 min at 145 °C, the IgE-binding capacity and the degranulation capacity of Ara h 2/6 were 600–700-fold lower than those in the native form [150]. More in vivo studies may be needed to validate the effects of non-enzymatic browning on the allergenicity of peanuts. Because most studies demonstrated the enhanced immunoreactivity of roasted peanuts, roasting might not be recommended as a method for allergenicity mitigation. In addition, more clinical studies with patients with a confirmed peanut allergy will be needed to compare the allergenicity of proteins in raw and roasted peanuts. The effects of boiling and thermal pressure treatment on the allergenicity of peanuts are discussed in Section 7.1 (Physical Treatment of Peanuts for Allergenicity Reduction) because boiled and autoclaved peanuts were consistently reported to have a significantly reduced allergenicity than raw peanuts.

## 7. Research Progress in Peanut Allergenicity Mitigation

To eliminate the allergenicity of a protein, many studies have been conducted to remove, modify, or mask peanut allergens or their epitopes to make them unable to bind to peanut-specific IgEs. It has been reported that certain processing approaches, such as enzymatic hydrolysis combined with roasting, high pressure treatment, ultrasound-assisted enzymatic treatment, or germination, can lead to a significant reduction in peanut immunoreactivity, offering promising techniques to mitigate the allergenicity of peanuts. Chemical and physical modifications can mask epitopes, thus reducing the allergenicity of peanut proteins. Protease hydrolysis can destroy epitopes to different degrees depending on the selectivity or specificity of the proteases used and hydrolysis conditions such as protease concentration, hydrolysis time, and pH, but may also produce peptides that still contain epitopes.

### 7.1. Physical Treatment of Peanuts for Allergenicity Reduction

#### 7.1.1. Thermal Processing

Boiling peanuts has been suggested as a method to reduce their allergenicity. The extensive boiling of peanuts has shown to significantly decrease IgE-binding activity and immunoreactivity; up to an 8- and 19-fold reduction in IgE-binding capacity was observed after 8 and 12 h of boiling, respectively [151]. This reduction in allergenicity is attributed to the transfer of water soluble low-molecular-weight allergens, such as Ara h 2, 6, and 7, from the peanut kernel into the cooking water during boiling [152,153]. Furthermore, the potential benefits of boiling peanuts have led to the exploration of using boiled peanuts in oral immunotherapy protocols for treating peanut allergies [154].

High-pressure methods, such as autoclaving, have been reported as effective novel techniques for reducing the allergenicity of peanuts [151]. Autoclaving at high pressures and temperatures has been shown to decrease major allergen detection and alter the protein secondary structure, leading to a reduction in intact allergenic proteins [155,156]. Autoclaving at extreme conditions (2.56 atm for 30 min) significantly decreased the IgE-binding capacity of peanut allergens, as demonstrated by in vitro and in vivo experiments [157,158]. While autoclaving can reduce IgE binding, it does not eliminate the allergen entirely. Further research is needed to determine the extent to which autoclaving can be used in peanut processing to reduce the allergenic potential of peanuts.

#### 7.1.2. Irradiation

Pulsed ultraviolet (PUV) light treatment is another physical method for reducing peanut allergenicity. Yang et al. treated protein extracts from raw for 2, 4, and 6 min and roasted peanuts and peanut butter slurry for 1, 2, and 3 min in a Steripulse XL-3000^®^ PUV system (Xenon Corporation, Woburn, MA, USA). The treatments resulted in a reduction in Ara h 1, Ara h 2, and Ara h 3 levels and decreased the IgE-binding ability by 12.9% to 6.7% [159]. The advantage of this method is fast and can be used for different forms of peanuts. However, peanuts are rich in unsaturated fat. Exposing peanuts to pulsed UV may accelerate lipid oxidation, which will result in rapid quality deterioration and harmful oxidation products [155]. Another drawback of using pulsed UV is that the solubility of peanut protein was significantly reduced after PUV treatment [160]. In addition to PUV treatment, gamma irradiation was also reported to reduce the IgG-binding of the whole peanut protein extract and Ara h 6 due to the significant changes in the secondary and tertiary structures of Ara h 6, particularly the loss of the α-helix structure [161]. However, reduced IgG-binding to an allergen does not necessarily mean reduced allergenicity, because IgG-binding is not a reliable indicator of allergy; reduced IgG-binding might implicate enhanced allergen tolerance by immunotherapy [162]. In addition, irradiation is not generally approved for peanuts, especially peanut butter, because it can cause rancidity in high-fat foods, leading to potential negative impacts on flavor and quality, rendering the product unacceptable to consumers.

### 7.2. Biological Methods of Peanuts for Allergenicity Reduction

#### 7.2.1. Conventional Breeding

The conventional breeding of low-allergenic peanuts involves identifying and selecting peanut varieties with naturally lower levels of allergenic proteins like Ara h 1, Ara h 2, Ara h 3, and Ara h 6. These varieties are then crossbred to combine the desired traits, creating new peanut lines with reduced allergenicity while maintaining desirable characteristics like yield and pest resistance [163]. Perkins et al. crossbred peanuts that were missing either an Ara h 2 or Ara h 3 isoform and produced a variety lacking both isoforms [164]. While conventional breeding has not yet yielded null mutants for allergens, naturally occurring genotypes deficient in Ara h 1 have been identified, indicating the potential for breeding hypoallergenic peanut varieties [165]. These methods offer high mutation rates and diversity in generating hypoallergenic peanuts without complex regenerative procedures. Although some natural genotypes deficient in certain allergens have been found, it is challenging to achieve completely null mutants, because the method’s progress is hindered by the complexity and diversity of peanut allergens.

#### 7.2.2. Irradiation Breeding

Irradiation breeding, using techniques like gamma irradiation and heavy-ion beam irradiation (HIBI), effectively reduces peanut allergenicity by altering protein structures and inducing stable gene mutations. Gamma irradiation lowers Th-2 lymphocyte activity and the antigenicity of allergens such as Ara h 6 [151,161], while heavy-ion beam irradiation (HIBI) produces knockout mutants lacking allergenic isoforms like Ara h 2 and Ara h 3 [166]. Studies have shown that irradiation breeding can yield peanut lines with reduced levels of advanced glycation end (AGE) products and lower IgE binding, indicating a potential role of specific protein subunits in the allergenicity of peanuts [167]. However, concerns about food safety due to potential chemical changes and regulatory challenges for irradiated foods must be addressed to ensure the safe production of allergen-reduced peanut products.

#### 7.2.3. Genetic Engineering

Genetic modification tools such as RNA interference (RNAi) and gene editing using CRISPR-Cas9 have been studied for reducing major allergenic proteins in peanuts. By using RNAi, researchers can target and degrade specific messenger RNA (mRNA) molecules that code for allergenic proteins, effectively reducing their production in the plant. Chu et al. silenced Ara h 2 and Ara h 6 by RNAi and produced three independent transgenic lines. All three lines were featured by significantly reduced Ara h 2 levels, whereas the level of Ara h 6 was only reduced in two lines. All three lines showed reduced binding to human IgE. In addition, there were no significant differences in the seed weight and germination rates between transgenic and non-transgenic plants [168]. Another study tried to produce hypoallergenic peanuts by silencing Ara h 1, Ara h 2, and Ara h 3 with RNAi and found that transgenic peanuts showed a 9%, 10%, and 16% reduction in Ara h 1, Ara h 2, and Ara h 3, respectively; 3% of transgenic seeds were free of all three allergens. The IgE-binding capacity was significantly reduced in at least nine transgenic seeds with lower Ara h 1 or Ara h 2 and Ara h 3 levels [169]. Gene editing technologies like CRISPR reduce peanut allergenicity by targeting and modifying the genes responsible for synthesizing major peanut allergens. Conner and colleagues successfully edited the 2S albumin allergen genes of peanuts by a multiplex gene editing strategy using CRISPR-Cas9 and two conserved guide RNAs, resulting in plant lines displaying the deletion of Ara h 2, Ara h 6, and Ara h 7 [170].

These studies highlight the potential of using genetic engineering tools such as RNAi, CRISPR/Cas9, and protein modification to reduce the allergenic potential of peanuts. However, genetic technology has two big drawbacks. One is the increasing repulsion to transgenic food by consumers. The other is that peanut allergens account for 20–30% of total peanut proteins, and if all the allergens are removed, peanuts may not taste like peanuts [169]. The CRISPR-Cas9 and other gene editing tools can sometimes modify unintended parts of the genome, leading to off-target effects that could have unforeseen consequences [171]. These significant barriers prevent the wide adoption of genetically engineered peanuts. Continued research and public education are crucial for navigating these challenges and realizing the full potential of genetic engineering in improving peanut safety.

#### 7.2.4. Enzymatic Treatment

The reported enzymatic approaches include hydrolysis of peanut protein with proteases and the enzymatic cross-linking with enzymes such as tyrosinase, peroxidase (POD), and transglutaminase (TGA). In vitro tests, animal studies, and human clinical studies confirmed that enzymatic hydrolysis/treatment effectively reduced the allergenic potential of peanuts, peanut flour, and peanut protein extract.

Protease hydrolysis breaks down large protein molecules into smaller peptides. This process can modify the primary structure of allergenic proteins, potentially disrupting the peanut-specific epitopes and reducing their ability to be recognized by the immune system’s allergen-specific IgE antibodies [172]. Proteases such as alpha-chymotrypsin, trypsin, Alcalase, bromelain, papain, Neutrase, and flavourzyme have shown different effectiveness in degrading major peanut allergens (Ara h 1, Ara h 2, Ara h 3, and Ara h 6) and reducing the IgE-binding of protein extracts from peanut kernels and peanut flour [172,173,174,175]. Cabanillas et al. discovered that 30 min Alcalase treatment resulted in an important decrease in Ara h 1, Ara h 2, and Ara h 3 levels and reduced IgE-binding reactivity by 98%; a 90 min Alcalase treatment could fully eliminate IgE-binding reactivity; while a 30 min flavourzyme treatment caused an increase in IgE reactivity, but 300 min led to a 65% inhibition of IgE binding [173]. Among all tested proteases, Alcalase, an endopeptidase preparation from *Bacillus licheniformis*, has displayed the highest efficacy in different studies because of its low specificity, which makes it less/non-selective and can cleave more peptide bonds [173,174,175]. Another study revealed that the hydrolysis of peanut proteins using papain, ficin, and bromelain at a concentration of 400 AzU/g also resulted in an 85–95% IgE-binding reduction in the soluble portion [176]. A human skin prick test revealed that treatment of peanut kernels with Alcalase significantly reduced the allergic response in children who were allergic to peanuts [177]. Studies using animal models (like BALB/c mice) have demonstrated that enzyme-treated peanuts lead to a decreased release of inflammatory factors and other markers associated with allergic reactions [178]. However, another study assessed the allergenicity of enzymatic hydrolyzed products using a different method and found that the allergenicity was retained after treating roasted peanut protein extract in a similar way [179]. In addition to the type of protease, the enzymatic treatment conditions, such as temperature, pH, enzyme concentration, treatment time, and pre-treatments, also affect the allergen contents and IgE binding of the hydrolyzed product [177,180]. Thus, optimizing enzymatic treatment conditions is important to achieve a high degree of desensitization no matter which enzyme is used. The above examples demonstrate that enzymatic hydrolysis/treatment is the most promising method to reduce the allergenicity of peanuts, peanut flour, or peanut protein extraction. However, more studies are needed to improve the allergenicity-reducing efficacy and to evaluate the impact of enzyme treatment on the sensory quality and consumer acceptance of peanuts and related products.

Enzyme catalyzed cross-linking has been reported to reduce food protein allergenicity by modifying the protein’s structure and masking or destroying the allergenic epitopes that bind to IgE antibodies; meanwhile, cross-linking also produces a high-molecular-weight polymer with reduced immunoreactivity and the ability to inhibit mast cell degranulation, which helps maintain T-helper (Th)1/Th2 immunobalance [181]. An early study found that POD treatment of roasted peanut extract resulted in a partial loss of Ara h 1 and Ara h 2, along with a reduced IgE-binding ability and the formation of new polymers; on the other hand, TGA treatment had no effect on the content of Ara h 1 and Ara h 2 as well as the IgE-binding ability; and both POD and TGA had no effect on the IgE-binding ability of the protein extract from raw peanuts [182]. In another study, the tyrosinases from *Agaricus bisporus* and *Trichoderma reesei tyrosinases* were used to cross-link peanut proteins, and the allergenicity of cross-linked products was tested by competitive ELISA, basophil activation tests, and in sensitized mice. The study concluded that the two tyrosinases increased the bioavailability of major peanut allergen Ara h 2 but did not significantly change the allergenic or tolerizing properties of the peanut protein extract because cross-linked proteins had preserved molecular and immunological features of peanut allergens [183]. It was also found that transglutaminase (TGA) cross-linking peanut protein hydrolysates had similar IgE-binding properties to the un-cross-linked hydrolysates, although it improved the emulsifying and foaming properties of the hydrolysate [166]. These examples indicate that enzyme-catalyzed cross-linking has limited effects on the allergenicity reduction in peanut proteins.

#### 7.2.5. Fermentation

In recent years, the potential of fermentation in reducing peanut allergenicity has been researched. Limited studies have shown that fermentation with *Bacillus natto*, lactic acid bacteria (LAB), and some edible molds can significantly reduce the IgE-binding properties of peanut proteins by potentially degrading allergens through the activity of enzymes produced during the process, increasing protein digestibility, and modulating the immune system [184,185,186]. The study of Pi and colleagues revealed that the autoclave-assisted fermentation with *Bacillus natto* reduced more than 77.3% of the IgE reactivity in raw peanut protein preparations because the process induced extensive proteolysis, unfolding, conformational changes, and a reduction in α-helices, which all affected epitope formation significantly [184]. In another study, peanut milk was co-fermented with *Lactobacillus plantarum strain P1* and *L. pentosus strain Y6*, resulting in the folding and degradation of allergenic proteins larger than 20 kDa, especially Ara h 1, during fermentation. In addition, co-fermentation improved the peanut milk flavor compared to the fermentation by *L. plantarum* or *L. pentosus* alone [185]. It was also reported that fermentation of peanut flour for up to 48 h using *Rhizopus oryzae* significantly degrades larger peanut proteins into smaller peptides but only slightly reduces IgE binding [186]. These studies indicate that, while promising, more research is needed to fully understand the long-term effects of fermentation on peanut allergenicity and to develop safe and effective fermentation strategies to produce hypoallergenic peanut products.

#### 7.2.6. Polyphenol–Protein Interactions

Polyphenols exist widely in plant food materials, including peanuts and peanut skins. They can reduce the allergenicity of peanut proteins through several mechanisms, including modifying the protein structure, promoting enzymatic digestion, and modulating immune responses. Polyphenols can bind to peanut proteins covalently or non-covalently, altering their structure and potentially masking the epitopes, thus reducing allergenicity. Non-covalent interactions can alter the protein’s secondary and tertiary structure, impacting its surface hydrophobicity and potentially reducing its ability to bind to IgE [187]. It was found that the peanut protein extract (PPE) covalently bound with the major green tea polyphenols EGCG and apple polyphenol chlorogenic acid (CA) under alkali conditions and resulted in a PPE-EGCG/CA conjugate with less folded structure, significantly reduced allergenicity in both in vitro and in vivo experiments, meanwhile significantly enhancing functional properties [188]. Sun and colleagues studied the ability of five major apple polyphenols (epicatechin, phlorizin, rutin, chlorogenic acid, and catechin) to reduce the allergenicity of Ara h 1 using a female BALB/c mice model. They found that polyphenol binding could alleviate the allergenicity of peanuts and regulate the MAPK-related signaling pathway. Among tested polyphenols, epicatechin binds to arginine in the epitopes of Ara h 1 and reduces the number of linear epitopes [189]. While beneficial effects on allergenicity are promising, the polyphenol–protein interactions can also impact other desirable properties like texture, flavor, and nutritional value. Most polyphenols can cause astringency and color changes to the food products, and some polyphenols inhibit the activity of digestive enzymes and reduce the digestibility of macronutrients and the absorption of some essential nutrients such as vitamins and minerals [155,190,191].

## 8. Management of Peanut Allergy

As discussed above, none of those approaches can completely eliminate peanut allergenicity, although some methods are more effective than others. Thus, proper management/treatment of peanut allergies remains essential, particularly for accidental exposures. Allergic reactions can range from mild to severe, including anaphylaxis, a life-threatening allergic reaction, based on the individual’s sensitivity and the amount of peanut protein ingested [192]. The management of peanut allergies involves strategies such as avoidance and carrying epinephrine auto-injectors for emergency treatment for severe reactions, although the risk of fatal anaphylaxis is low [70]. Early introduction of peanut-containing products into the diets of high-risk infants has been recommended to prevent peanut allergies [59,193]. The introduction of peanuts before 12 months of age is advised for infants with severe eczema and/or egg allergies to reduce the occurrence of peanut allergies in countries with high peanut consumption [194]. In addition, oral immunotherapy (OIT) has shown promise in improving the health-related quality of life among patients [195]. Peanut OIT is a medical treatment. It involves gradually exposing a patient to increasing amounts of peanut proteins under strict medical supervision of an allergist to build tolerance [196]. However, great care must be taken when OIT is used for children with peanut anaphylaxis. One study investigated the efficacy and safety of peanut OIT in 23 children with a confirmed peanut allergy and found that 2.6% of children displayed mild to moderate side effects, while 1.3% of children exhibited pulmonary obstruction symptoms [197]. In addition to the above management strategies, food product labeling plays a crucial role in peanut allergy management by enabling individuals with peanut allergies to identify and avoid potential allergenics. Clear and accurate labeling is essential for preventing accidental ingestion and severe allergic reactions [198,199]. Of course, consumers with a peanut allergy are responsible for reading the label carefully.

## 9. Conclusions and Future Perspectives

This review depicts that peanut allergenic proteins are highly diverse, and their amino acid sequence and composition are quite different, particularly those in different protein super families, which has significant impacts on the chemical characteristics such as heat and digestive stability, solubility, and allergenicity. The peanut allergens in the same protein family have some similarities in amino acid composition/sequence, solubility, and allergenicity, but allergens in different protein families displayed distinct differences in their solubility and allergenicity. The allergenicity of peanut proteins are determined by the number of epitopes in their amino acid sequences, and their configurations such as secondary and tertiary structures, which are affected by the environmental factors such as temperature, pH, and the presence of other compounds such as polyphenols. Many studies have been conducted to explore efficient methods/strategies to eliminate or reduce peanut allergenicity. These methods include thermal treatments like boiling and autoclaving, irradiation, conventional breeding, irradiate breeding, genetic engineering, enzymatic hydrolysis, fermentation, and protein–polyphenol interaction/conjugation. Among these methods, enzymatic hydrolysis with proteases displayed the highest efficacy in degrading the native and denatured allergenic proteins and reducing IgE binding and is considered the most promising method for producing hypoallergenic peanut products. However, none of the discussed methods can completely eliminate the allergenic potential of all allergens in peanuts. Some methods may have negative impacts on peanut quality and safety. Strategies combining more than one method may be needed to achieve a higher allergenicity reduction. Thus, it is still imperative to practice peanut allergy management strategies including avoidance, prevention of accidental exposure, and tolerance induction and treatments. As aforementioned, more research is needed to identify the epitopes of peanut oleosins, defensins, and nsLTPs for better understanding their allergenicity. In addition, the allergenicity of some peanut proteins were only evaluated by an IgE-binding test; clinical research could provide confirmative information. In terms of the methods for reducing peanut allergenicity, it is essential to conduct double-blind clinical studies to validate if the peanut products resulted from different treatments are really hypoallergenic. There is also a need to evaluate the feasibility and cost of effectiveness of allergenicity-reducing methods. Furthermore, it is also crucial to investigate the impacts of different allergen-reducing methods on the safety, nutritional, and sensory quality, as well as the oxidative stability of peanuts and peanut-derived products.

## Figures and Tables

**Table 1:** Amino acid compositions of peanuts and peanut-derived products [10].

Amino Acid (AA)	Raw Peanuts	Roasted Peanuts (Dry Roasted)	Peanut Flour (22% Fat)	Peanut Flour (Fat Free)	Soybean (Dry Roasted)
Tryptophan	0.25	0.23	0.328	0.507	0.575
Threonine	0.883	0.811	1.16	1.79	1.72
Isoleucine	0.907	0.833	1.19	1.84	1.92
Leucine	1.67	1.54	2.19	3.38	3.22
Lysine	0.926	0.85	1.21	1.87	2.63
Methionine	0.317	0.291	0.415	0.641	0.534
Cystine	0.331	0.304	0.433	0.669	0.638
Phenylalanine	1.38	1.23	1.75	2.7	2.07
Tyrosine	1.05	0.963	1.37	2.12	1.5
Valine	1.08	0.993	1.42	2.19	1.98
Arginine	3.08	2.83	4.04	6.24	3.07
Histidine	0.652	0.599	0.854	1.32	1.07
Alanine	1.02	0.941	1.34	2.08	1.86
Aspartic acid	3.15	2.89	4.12	6.37	4.98
Glutamic acid	5.39	4.95	7.06	10.9	7.67
Glycine	1.55	1.43	2.04	3.14	1.83
Proline	1.14	1.04	1.49	2.3	2.32
Serine	1.27	1.17	1.66	2.57	2.29
Total AA	26.46	23.895	34.07	52.627	41.877

**Table 2:** Prevalence of peanut allergies across different countries and regions (self-reported or parent-reported).

Country	Sample Size	Age Group	Prevalence (%)	Reference
Australia	Various	Infants	about 3.0%	[41]
Australia	7290	Infants	2.6%	[42]
United States	51,819	Children Adults	2.2% 1.8%	[34,43]
United Kingdom	5171 1303	Children Children	1.85% 2.5%	[36,44]
Netherlands	1421	2-year-olds	1.69%	[39]
Germany	13,300	Adults	ahout 1.1%	[45]
Europe region		Whole population	1.0–2.1	[46]
Italy	about 6900 visits	Various	0.7%	[47]
Israel	4657	Schoolchildren	0.17%	[36]
China	138,740	Various	0.2–0.3%	[48,49]
Hong Kong	7393	14 years old or younger	0.3–0.5%	[50]
Taiwan	16,200	All age groups	1.3% in children, 1.6% in adolescents, and 0.9% in adults	[51]
Turkey	534 food allergy patients 10,096	13–18 years old (preschooler, school age, and adolescents) Adolescents	14.9% of food-allergic population 0.05% (IgE confirmed)	[52,53]
Saudi Arabia	15,142 2130	Various Children 5–18 years	4% 4%	[54,55]
South Africa	Various	Children	<3%	[56]
Thailand	Various	6 months–7 years	Very low	[57]
Cuba	316 food-allergic patients	Adults and children	4.6% in adult population 18.6% in food-allergic adults 25.8% in food-allergic children	[58]

**Table 3:** Officially registered peanut allergens [75].

Allergen	Biochemical Name	Molecular Weight (SDS-PAGE)	Amino Acid Length *	GenBank Nucleotide	GenBank Protein
Ara h 1.0101 Ara h 1.0101	Cupin (vicillin-type, 7S globulin)	64 kDa	626	L34402	AAB00861 P43238
Ara h 2.0101 Ara h 2.0201	Conglutin (2S albumin)	17 kDa (16.67 and 18.05 kDa)	156 172	AY007229 AY158467	AAK96887 AAN77576
Ara h 3.0101 Ara h 3.0201	Cupin (legumin-type, 11S globulin, and glycinin)	60 kDa, 37 kDa (fragment)	507 530	AF093541 AF086821	AAC63045 AAD47382
Ara h 4 (Ara h 3.0201)	Renamed to Ara h 3.02	Same as Ara h 3			
Ara h 5	Profilin	15 kDa (14.051 kDa)	131	AF059616	AAD55587
Ara h 6	Conglutin (2S albumin)	15 kDa	129	AF092846	AAD56337
Ara h 7.0101 Ara h 7.0201 Ara h 7.0301	Conglutin (2S albumin)	15 kDa 17.374 kDa 17.3 kDa	131 164 158	AF091737 EU046325 AY722691	AAD56719 ABW17159 AAU21496
Ara h 8.0101 Ara h 8.0201	Pathogenesis-related protein, PR-10, and Bet v 1 family member	17 kDa 16.9 kDa	158 157	AY328088 EF436550	AAQ91847 ABP97433
Ara h 9.0101 Ara h 9.0201	Non-specific lipid-transfer protein type 1	9.8 kDa	116 92	EU159429 EU161278	ABX56711 ABX75045
Ara h 10.0101 Ara h 10.02.01	Oleosin	16 kDa 15.4 kDa	169 150	AY722694 AY722695	AAU21499 AAU21500
Ara h 11.0101 Ara h11.0201	Oleosin	14 kDa	137 137	DQ097716	AAZ20276 AAZ20277
Ara h 12	Defensin	8 kDa (reducing), 12 kDa (non-reducing), and 5.184 kDa (mass)	-	EY396089	
Ara h 13	Defensin	8 kDa (reducing), 11 kDa (non-reducing), and 5.472 kDa (mass)	-	EY396019 EE124955	
Ara h 14 (3 isomers)	Oleosin	17.5 kDa	176	AF325917 AF325918 AY605694	AAK13449 AAK13450 AAT11925
Ara h 15	Oleosin	17 kDa	176	AY722696	AAU21501
Ara h 16	Non-specific lipid transfer protein type 2, nsLTP-2	8.5 kDa by SDS PAGE, reducing	166	KX592166	ASU04353
Ara h 17	Non-specific lipid transfer protein type 1, nsLTP-1	11 kDa by SDS-PAGE, reducing	93	KX592165	ASU04352
Ara h 18	Cyclophilin, peptidyl-prolyl cis-trans isomerase	21 kDa	172	XM_025819515	XP_025675300

* The lengths of the amino acid chains of each allergen and its isomers are calculated or counted from their amino acid sequences through the GenBank Protein.

**Table 4:** Number of individual amino acids in peanut allergens.

AA	Ara h 1	Ara h 2	Ara h 3	Ara h 4	Ara h 5	Ara h 6	Ara h 7	Ara h 8	Ara h 9	Ara h 10	Ara h 11	Ara h 12	Ara h 13	Ara h 14	Ara h 15	Ara h 16	Ara h 17	Ara h 18
A (Ala)	33	11	32	32	8	5	11	11	20	15	17	7	2	14	20	5	9	11
C (Cys)	7	8	4	8	2	10	6	0	9	1	0	9	9	1	1	8	8	4
D (Asp)	30	9	25	24	7	9	8	9	1	6	2	3	5	9	8	0	1	7
E (Glu)	68	14	50	50	8	11	15	16	0	4	3	3	5	1	1	1	0	9
F (Phe)	22	3	23	31	3	3	2	5	4	6	5	6	6	4	5	0	2	12
G (Gly)	45	7	36	40	18	9	10	17	12	20	17	4	6	19	28	5	9	25
H (His)	15	3	10	13	3	3	1	2	0	4	2	4	3	3	1	0	0	4
I (Ile)	25	3	19	20	13	3	2	12	5	9	8	1	5	12	13	2	9	9
K (Lys)	36	3	9	8	6	2	2	18	5	8	5	6	8	6	6	3	7	10
L (Leu)	43	17	33	37	11	7	15	10	11	16	17	5	8	17	14	6	4	7
M (Met)	8	4	0	1	5	8	4	2	5	4	2	2	2	5	2	0	0	5
N (Asn)	41	7	39	38	4	7	7	6	6	2	1	4	3	1	1	3	7	8
P (Pro)	38	8	29	30	9	3	10	9	8	8	6	1	1	10	7	9	5	7
Q (Gln)	43	23	44	45	7	19	21	1	3	9	9	1	2	11	4	2	4	6
R (Arg)	59	17	56	51	1	13	20	1	5	7	6	1	5	9	6	4	1	8
S (Ser)	45	11	34	34	6	9	10	6	8	10	6	2	3	12	16	8	15	12
T (Thr)	21	1	15	14	8	2	2	13	6	20	13	5	1	20	13	5	6	12
V (Val)	33	2	27	28	5	4	9	12	7	16	13	5	3	19	8	5	4	13
W (Trp)	5	1	3	5	2	0	3	0	0	1	1	1	1	1	1	0	0	1
Y (Tyr)	9	4	19	20	5	2	2	7	1	3	4	1	1	2	11	2	2	2
**SUM**	**626**	**156**	**507**	**529**	**131**	**129**	**160**	**157**	**116**	**169**	**137**	**71**	**79**	**176**	**166**	**68**	**93**	**172**

**Table 5:** Stability and allergenicity of different groups of peanut proteins.

Allergen Groups	Allergens	Stability	Allergenic Potential	Reference
Cupins	Ara h 1, Ara h 3	Stable to heat but less stable to digestion	High	[78,108,129]
2S Albumins	Ara h 2, Ara h 6, and Ara h 7	Extremely stable to heat and gastric digestion	Very High	[108,112,115,130]
Profilin	Ara h 5	Stable	Moderate to High	[119]
PR-10 Protein	Ara h 8	Unstable to heat and digestion	Moderate	[120]
Oleosins	Ara h 10, 11, 14, and 15	Highly stable	High	[99]
Non-specific lipid transfer proteins (nsLTPs)	Ara h 9, Ara h 16, and Ara h 17	Highly resistant to heat and digestion	Moderate	[86]
Peanut defensins	Ara h 12, Ara h 13	Resistant to heat and digestion	Low	[100]
Cyclophilin	Ara h 18	Not fully elucidated	Low	[104,123]

## Data Availability

No new data were created or analyzed in this study. Data sharing is not applicable to this article.
